# Sinomenine Hydrochloride Promotes TSHR-Dependent Redifferentiation in Papillary Thyroid Cancer

**DOI:** 10.3390/ijms231810709

**Published:** 2022-09-14

**Authors:** Jing Zhang, Aomei Zhao, Xi Jia, Xinru Li, Yiqian Liang, Yan Liu, Xin Xie, Xijie Qu, Qi Wang, Yuemin Zhang, Rui Gao, Yan Yu, Aimin Yang

**Affiliations:** 1 Department of Nuclear Medicine, The First Affiliated Hospital of Xi’an Jiaotong University, Xi’an 710061, China; 2 Department of Public Health, Health Science Center of Xi’an Jiaotong University, Xi’an 710061, China

**Keywords:** sinomenine hydrochloride, redifferentiation, sodium/iodide symporter, radioactive iodine uptake, papillary thyroid cancer

## Abstract

Radioactive iodine (RAI) plays an important role in the diagnosis and treatment of papillary thyroid cancer (PTC). The curative effects of RAI therapy are not only related to radiosensitivity but also closely related to the accumulation of radionuclides in the lesion in PTC. Sinomenine hydrochloride (SH) can suppress tumor growth and increase radiosensitivity in several tumor cells, including PTC. The aim of this research was to investigate the therapeutic potential of SH on PTC cell redifferentiation. In this study, we treated BCPAP and TPC-1 cells with SH and tested the expression of thyroid differentiation-related genes. RAI uptake caused by SH-pretreatment was also evaluated. The results indicate that 4 mM SH significantly inhibited proliferation and increased the expression of the thyroid iodine-handling gene compared with the control group (*p* < 0.005), including the sodium/iodide symporter (NIS). Furthermore, SH also upregulated the membrane localization of NIS and RAI uptake. We further verified that upregulation of NIS was associated with the activation of the thyroid-stimulating hormone receptor (TSHR)/cyclic adenosine monophosphate (cAMP) signaling pathway. In conclusion, SH can inhibit proliferation, induce apoptosis, promote redifferentiation, and then increase the efficacy of RAI therapy in PTC cells. Thus, our results suggest that SH could be useful as an adjuvant therapy in combination with RAI therapy in PTC.

## 1. Introduction

In recent decades, the incidence of thyroid cancer has substantially increased globally, largely driven by increases in papillary thyroid cancer (PTC) [1]. The vast majority of patients with PTC have a good prognosis after surgery, radioactive iodine (RAI) therapy, and thyroid stimulating hormone (TSH) suppression therapy [2]. In China, the five-year survival rate of differentiated thyroid carcinoma (DTC) patients can reach 84.3%, with a rate of 98.7% in developed countries such as the United States [3]. However, some patients gradually dedifferentiate under natural conditions or during treatment and lose the ability to uptake iodine, ultimately, resulting in resistance to RAI therapy [4], with a ten-year survival rate less than 10% [5]. The gap in survival rates and the occurrence of radioiodine resistance have attracted our attention. RAI therapy is often regarded as a key treatment for thyroid cancer, relying on the unique function of thyroid follicular cells to absorb and concentrate iodide. This process involves several key thyroid–iodine handling genes [6,7], including sodium/iodide symporter (NIS), thyroglobulin (TG), thyroid peroxidase (TPO), thyroid-stimulating hormone receptor (TSHR) and thyroid-specific transcription factors, such as paired box gene-8 (PAX-8). NIS is normally expressed in the basal membrane of follicular thyroid cells, which transports iodide ions into the cell in an active transport manner [6,8]. TG and TPO are mainly involved in the activation and iodization of iodide. Thyroid transcription factor PAX8 is involved in the regulation of thyroid genes. By acting on the TSHR in the thyroid cell membrane, TSH increases the expression of many of these iodide-handling genes in thyroid cells [9].

The mechanisms of resistance to RAI are based on the aberrant silencing of the expression of iodine-metabolizing genes [6], which is caused by acquired point mutations, chromosomal rearrangement, and aberrant gene methylation [10]. BRAF^V600E^ mutation is one of the most common genetic aberrations in PTC [11], which activates the mitogen-activated protein kinase (MAPK) signaling pathway and is associated with an increased risk of metastasis and recurrence, resistance to RAI therapy, and poor prognosis [12,13]. At present, targeted therapies have emerged as a promising alternative for the treatment of these patients [14]. However, primary or acquired drug resistance and chronic adverse effects lead to unsatisfactory clinical prognosis [15]. Accordingly, further studies are needed to increase RAI uptake via redifferentiation induction.

Sinomenine is a pure alkaloid extracted from the roots and stems of sinomenium acutum, and its water-soluble form sinomenine hydrochloride (SH) has been effectively used in clinical practice in China to treat rheumatoid arthritis with minimal side-effects [16,17]. In addition, SH also plays a vital role in anticancer [18]. Recently, several studies have confirmed the potential of SH as a radiosensitizer [16,19], and in particular, our group previous study has demonstrated that SH can synergistically increase the efficacy of RAI on PTC in vitro [20]. To our knowledge, the therapeutic effect of RAI is associated with not only radiosensitivity but also with the uptake of iodine in the thyroid lesion [21]. Studies have also confirmed this notion. Some traditional Chinese medicine (TCM) extracts, such as curcumin [22,23] and flavonoid quercetin [24], do not only enhance radiosensitivity in thyroid cancer but also promote cell redifferentiation and increase the uptake of RAI. To date, the redifferentiation ability of SH has not been investigated in PTC.

We focused on BCPAP and TPC-1 cells to explore the role of redifferentiation in SH. We demonstrated that SH could effectively inhibit PTC cell proliferation, induce apoptosis, and promote redifferentiation. Furthermore, we figured out that SH activated TSHR/cyclic adenosine monophosphate (cAMP) signaling pathways to increase the expression and plasma membrane localization of NIS and ultimately, promote the uptake of RAI. Combined with the previous findings of our research group, SH does not only increase the radiosensitivity of DTC but also induces redifferentiation in DTC cells. This makes it possible to concentrate more on RAI and how it can play a more effective role in radiation injury. The role of SH in DTC provides a new therapeutic opportunity for iodine refractory patients and is also a potential therapeutic method to reduce the therapeutic dose of RAI in DTC patients and reduce the adverse effects of treatment.

## 2. Results

### 2.1. SH-Inhibited Cell Proliferation and Induced Cell Apoptosis in BCPAP and TPC-1 Cells

**Figure 1:**
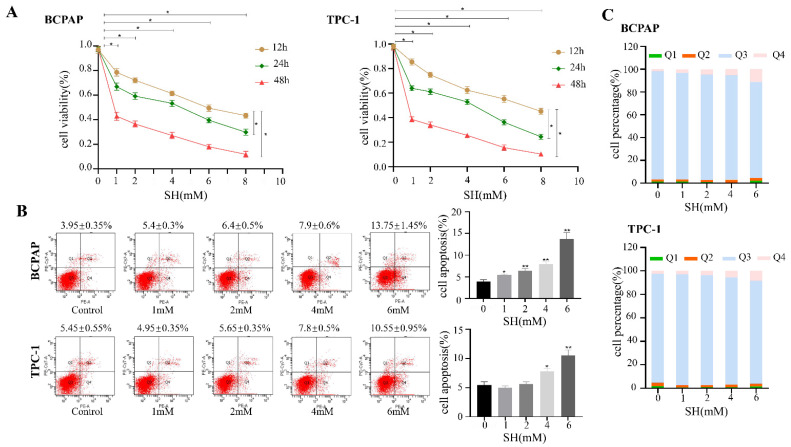
SH-inhibited cell proliferation and induced cell apoptosis in thyroid cancer cells. (**A**) To evaluate the effect of SH on cell proliferation, different thyroid cancer cell lines were treated with different doses (0, 1, 2, 4, 6, 8 mM) of SH for the indicated times (12 h, 24 h, 48 h). (**B**) Cell apoptosis was evaluated using flow cytometry when the indicated cells were treated with SH at different concentrations (0, 1, 2, 4, 6 mM) for 24 h. Apoptosis (%) represents the sum of early apoptotic and late apoptotic cells. (**C**) The percentage of live, early, and late apoptotic and necrotic cells. Q1: necrotic cells; Q2: late apoptotic cells; Q3: live cells; Q4: early apoptotic cells. The data are presented as the means ± SD of three independent experiments which are expressed as fold increase over control. * *p* < 0.05. ** *p* < 0.005. SH: sinomenine hydrochloride.

We performed a CCK-8 assay to determine the effect of SH on cell proliferation with different concentrations of SH (0, 1, 2, 4, 6, 8 mM) for 12 h, 24 h, and 48 h. As shown in Figure 1A, SH treatment significantly inhibited cell proliferation in BCPAP and TPC-1 cells in a time- and dose-dependent manner (*p* < 0.05). 

To study the effect of SH on apoptosis, cells were treated with SH at different concentrations (0, 1, 2, 4, 6 mM) for 24 h, respectively. In BCPAP cells, SH induced apoptosis in a dose-dependent manner (*p* < 0.05, Figure 1B). However, in TPC-1 cells, SH did not effectively inhibit apoptosis at low concentrations (1 mM and 2 mM). When the concentration of SH reached 4 mM, SH increased the apoptosis rate of TPC-1 cells, and the results were statistically significant (*p* < 0.05, Figure 1B). Cell apoptosis (%) represents the sum of early apoptotic and late apoptotic cells. The percentage of live, early, and late apoptotic and necrotic cells are shown in Figure 1C.

### 2.2. SH-Promoted Thyroid Iodine-Handling Gene Expression in BCPAP and TPC-1 Cells

**Figure 2:**
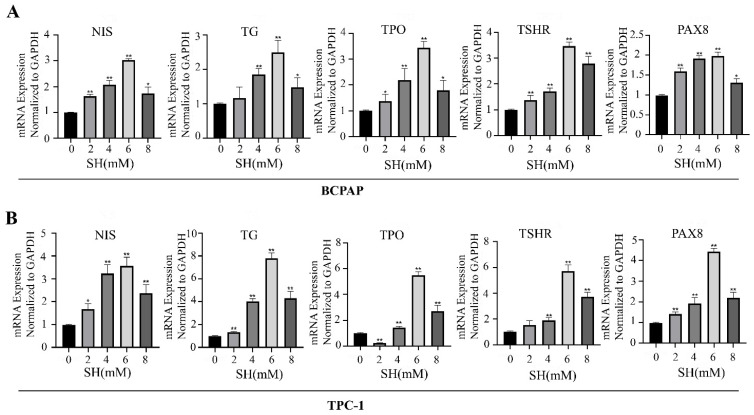
SH-promoted cell redifferentiation in BCPAP and TPC-1 cells. The BCPAP (**A**) and TPC-1 (**B**) cells were exposed to fixed concentrations of SH (0, 2, 4, 6, 8 mM) for 24 h. SH upregulated the expression of thyroid-specific genes and transcription factor expression in BCPAP and TPC-1 cells. Data are the mean ± SD of three samples per group. * *p* < 0.05 vs. control. ** *p* < 0.005 vs. control. NIS: sodium/iodide symporter; TG: thyroglobulin; TPO: thyroid peroxidase; TSHR: thyroid stimulating hormone receptor; PAX-8: paired box gene-8.

To assess the effect of SH in inducing redifferentiation in BCPAP and TPC-1 cells, the expressions of thyroid-specific genes such as NIS, TG, TPO, TSHR, and transcription factors PAX8 were detected using RT-qPCR. As shown in Figure 2, the expression of thyroid-specific genes and the transcription factor in SH treatment groups significantly increased to various extents compared with the control group. In particular, the SH treatment concentration of 6 mM showed the highest degree of redifferentiation (The upregulation multiples are as follows: NIS: 3.04 ± 0.05 and 3.57 ± 0.38, TG: 2.5 ± 0.36 and 7.8 ± 0.45, TPO: 3.45 ± 0.23 and 5.48 ± 0.29, TSHR: 3.46 ± 0.16 and 5.71 ± 0.48, PAX8: 1.97 ± 0.1 and 4.44 ± 0.15), respectively, in BCPAP and TPC-1 cells (all *p* < 0.005). However, increased TSHR expression after 2 mM SH treatment in TPC-1 exerted no significant effects compared with the control (Figure 2B). The TPO gene showed decreased expression after treatment with 2 mM SH in TPC-1 cells (*p* < 0.005, Figure 2B).

### 2.3. SH Upregulated the Expression and Plasma Membrane Localization of NIS and Increased RAI Uptake in BCPAP and TPC-1 Cells

**Figure 3:**
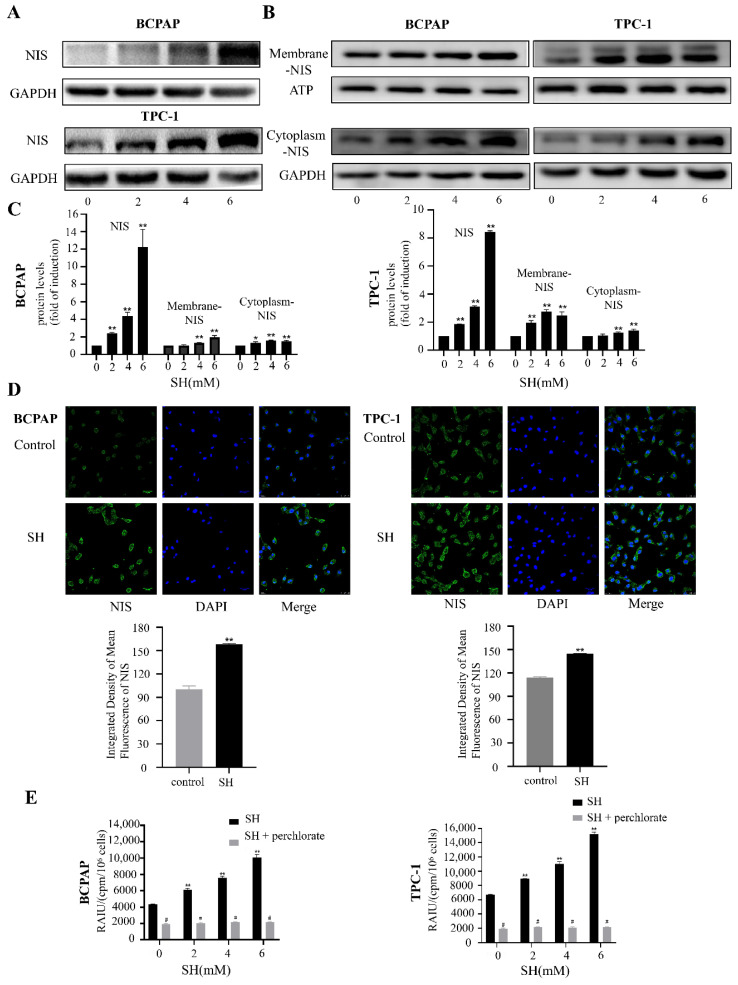
SH upregulated expression and plasma membrane localization of NIS and increased RAI uptake in BCPAP and TPC-1 cells. (**A**,**B**) Western blot analysis shows the expression of NIS in whole cell lysates (**A**), cell membrane (**B**), and cytoplasmic proteins (**B**) after 48 h with SH treatment. GAPDH and ATP were used as the loading control. (**C**) Quantitative analysis of band intensities on Western blots. (**D**) Immunofluorescence of NIS protein in BCPAP and TPC-1 cells treated with 4 mM SH for 48 h was detected using confocal microscope with high magnification fields (63×). Scale bar represents 25 μm. (**E**) BCPAP and TPC-1 cells were treated with fixed concentrations of SH (0, 2, 4, 6 mM) for 48 h, followed by ^125^I uptake assays. In addition, KClO4 was used as a competitive inhibitor of iodide transport. The cells were preincubated with 300 μM KClO4 for 30 min to inhibit ^125^I uptake, followed by the ^125^I uptake test. Data are presented as mean ± SD of values. * *p* < 0.05 vs. control. ** *p* < 0.005 vs. control. ^#^ *p* < 0.05 vs. relevant group treated by SH only. RAIU: RAI uptake.

RAI accumulation in thyroid cells is mainly mediated by the NIS protein. Therefore, we evaluated the protein expression level of NIS after SH treatment for 48 h. As shown in Figure 3A,C, the expression of NIS protein significantly increased compared with the control group. The immunofluorescence results also indicate that NIS protein expression increased after SH treatment for 48 h (*p* < 0.005, Figure 3D).

In previous studies, the immunohistochemistry of thyroid cancer samples revealed that NIS is clearly expressed or even overexpressed in most thyroid cancer cells, but there was a phenomenon of the impaired membrane targeting of NIS and insufficient retention of NIS in the membrane [25,26]. Considering that the therapeutic effect of RAI ultimately depends on the expression of NIS on the plasma membrane of thyroid cancer cells, we extracted cell membrane proteins and cytoplasmic proteins to study the expression of NIS in thyroid cancer cells. Cells were treated with SH for 48 h. As shown in Figure 3B,C, both the NIS membrane and cytoplasmic proteins increased to various degrees after SH treatment.

To determine whether RAI accumulation increased together with the upregulation of NIS function after SH treatment, we performed an ^125^I uptake assay. SH treatment caused a significant increase in the RAI uptake capacity in BCPAP and TPC-1 cells (Figure 3E). To determine the association between the increase in RAI uptake and SH-mediated NIS function, we performed an inhibition study with a specific inhibitor of NIS, KClO4 (300 μM), for 30 min. The enhanced RAI uptake was inhibited by KClO4. These results suggest that treatment with SH increased the functional expression of NIS protein similar to the mRNA expression levels and increased RAI uptake in BCPAP and TPC-1 cells.

### 2.4. SH Increased NIS-Mediated RAI Uptake by the Activation of the TSHR/cAMP Signaling Pathway in BCPAP and TPC-1 Cells

**Figure 4:**
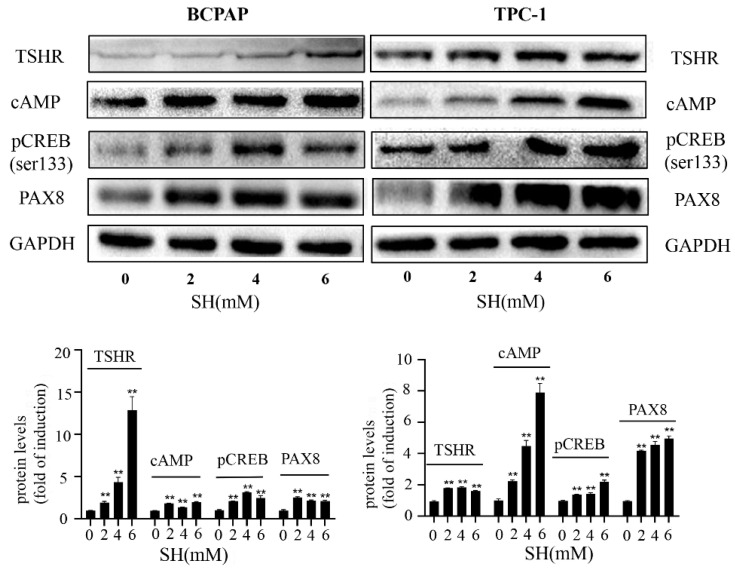
SH activated TSHR/cAMP/CREB/PAX8 signaling pathway in BCPAP and TPC-1 cells. The expressions of TSHR, cAMP, pCREB (ser 133), and PAX8 in BCPAP and TPC-1 cells were determined by Western blot with quantitative analysis. Cells were treated with SH for 48 h. GAPDH was used as the loading control. ** *p* < 0.005 vs. control. cAMP: cyclic adenosine monophosphate; CREB: phosphorylation of cAMP response element-binding protein.

**Figure 5:**
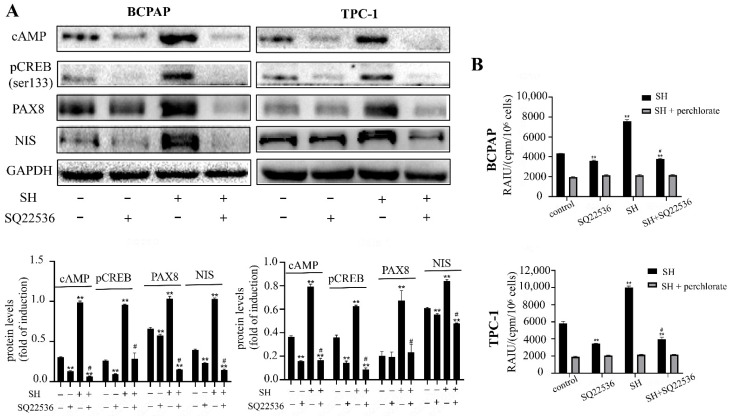
SH increased NIS-mediated RAI uptake by activation of TSHR/cAMP/CREB/PAX8 signaling pathway in BCPAP and TPC-1 cells. (**A**) The expressions of cAMP, pCREB, PAX8, and NIS in BCPAP and TPC-1 cells treated with or without 4 mM SH and SQ22536 for 48 h were determined by Western blot and quantitatively analyzed. (**B**) RAIU in BCPAP and TPC-1 cells was determined in treated cells with or without 4 mM SH and SQ22536 for 48 h. KClO4 was used as a selective inhibitor for NIS-mediated iodide uptake. ** *p* < 0.005 vs. control. ^#^ *p* < 0.05 vs. relevant group treated by SH only.

We have confirmed that SH can upregulate the expression of TSHR mRNA and promote the uptake of RAI. Numerous reports have demonstrated that activation of the TSHR is critical for optimal NIS expression and localization to plasma membrane [21], which affects the uptake of RAI. TSHR is preferentially coupled to G-alpha proteins upon TSH binding, leading to the activation of adenylyl cyclase and an increase in cAMP [27,28]. cAMP then stimulates the signaling pathway of the NIS upstream enhancer (NUE) by activating the Pax8 binding site or cAMP response element-binding protein (CREB), which, in turn, induces NIS transcription [26,29]. Hence, we explored whether the activation of the TSHR/cAMP pathway may play a role in an NIS-mediated RAI uptake increase. As shown in Figure 4, the protein level of TSHR, cAMP, pCREB (Ser133), and PAX8 were remarkably increased by SH treatment for 48 h in BCPAP and TPC-1 cells. In order to further investigate the role of TSHR in the redifferentiation process, thyroid cells were treated with SQ22536, a specific cAMP inhibitor. As shown in Figure 5A, the cAMP protein level and its downstream proteins (pCREB and PAX8) were successfully inhibited by SQ22536. An SH-induced increase in RAI uptake was also inhibited by SQ22536 (Figure 5B). Taken together, these data suggest that SH promoted the redifferentiation and increased the RAI uptake of thyroid tumor cells by upregulating the TSHR/cAMP signaling pathway in BCPAP and TPC-1 cells.

## 3. Discussion

RAI therapy is one of the critical treatments for medium- and high-risk differentiated thyroid cancer. Effective RAI uptake is an important element of RAI therapy and associates with the unique function of the iodide-metabolizing machinery of thyroid follicular cells, especially the expression and plasma membrane localization of NIS [30]. Compared with normal thyroid cells, the expression of NIS and uptake of RAI in PTC cells are downregulated to some extent. The loss of iodide uptake ability makes the tumor resistant to RAI therapy and eventually, it develops into radioiodine refractory differentiated thyroid cancer (RAIR-DTC) [26]. Activation of MAPK/extracellular signal-regulated protein kinase (ERK) and phosphoinositide 3-kinase (PI3K)/serine–threonine kinase (AKT) were reported to be related to the dedifferentiation and decrease in NIS expression in DTC [31,32], thus resulting in poor responsiveness to RAI therapy. Many attempts have been made to target MAPK and PI3K signaling to increase iodide avidity using various kinase inhibitors such as selumetinib [33], vemurafenib [34], dabrafenib [35], and LY294002 [36] in pre-clinical and clinical situations. However, relevant studies of these agents in inducing redifferentiation and increasing iodine uptake are still limited. Therefore, more research is also needed to find better treatment options. 

TCM has played an increasing role in tumor research in recent years. Sinomenine has been used for centuries in the treatment of patients with autoimmune diseases as it possesses immunosuppressive and anti-inflammatory activities [17]. The effect of SH on the differentiation of mouse preosteoblastic cell lines and dendritic cells [37,38] has been verified, but its role in thyroid cancer is still rare. Moreover, some TCM components such as curcumin have made remarkable achievements in the field of redifferentiation [22]. Previous studies from our group confirmed that SH can synergistically increase the radiation injury effect of RAI [20]. On this basis, we committed to further study of the redifferentiation role of SH in thyroid cancer cells. Here, we found that the treatment of cell lines with SH can increase the expression of thyroid differentiation-related genes (including the normal thyroid cell line Htori3 (Figure S1)) and increase the uptake of RAI. Considering that RAI treatment is mainly performed after surgery, follow-up studies have not delved into the study of normal thyroid cells. Notably, as BRAF mutant thyroid cancers are generally known for their insensitivity to RAI therapy [39] and SH treatment resulted in a 1.747-fold increase in iodine uptake by the BRAF mutant cell line BCPAP (4 mM SH), this further strengthens the clinical potential of SH in restoring RAI sensitivity, even in poorly differentiated thyroid cancers.

It is well known that TSH is one of the major regulators of NIS expression in the thyroid [40]. High levels of TSH are essential for the ablation of residual tissue in thyroid cancer, which can promote the membrane localization of NIS and is a key step in RAI uptake [41]. After TSH binds to TSHR, adenylyl cyclase is stimulated by the Gs protein, causing the production of cAMP [27]. Then, cAMP induces NIS transcription followed by stimulating NUE activity through activating several signaling pathways [21,26]. The literature reported that nevirapine induced redifferentiation and RAI uptake via the TSHR/cAMP/CREB/PAX8 signal pathway in dedifferentiated thyroid cancer [42]. Our study also showed that cAMP and pCREB (Ser133) were upregulated in response to SH. When cAMP was inhibited, the role of SH in inducing NIS upregulation and the increase in RAI uptake was blocked up because of an inhibition of pCREB (Ser133) and PAX8. These results suggest that SH increased NIS-induced RAI uptake via activation of the TSHR/cAMP signal pathway in BCPAP and TPC-1 cells.

In addition to regulating the TSHR/cAMP signaling pathway, other pathways may also be involved in the redifferentiation of thyroid cancer by SH. It was found that sinomenine can inhibit growth and invasion through the MAPK/ERK and PI3K/Akt/mTOR pathways in multiple tumor cells [43,44,45]. In PTC cells, we noticed that SH can inhibit proliferation and induce apoptosis, therefore, it is reasonable for us to speculate that SH might similarly suppress the activation of MAPK and PI3K signaling pathways in thyroid cancer cells. It has been reported that targeted inhibition of MAPK [6,33] or PI3K [46,47] pathways can induce the expression of NIS and restore the uptake of RAI by thyroid cancer cells. Thus, the differentiating effects of SH are perhaps associated with the MAPK and PI3K signaling pathways. However, we found that SH could not effectively inhibit MAPK and PI3K phosphorylation (Figure S2), and whether these two signaling pathways are involved in the induced redifferentiation process of SH remains to be further explored.

The success of RAI therapy for thyroid cancers depends not only on the uptake of RAI but also on the radiosensitivity to RAI. Our group previously demonstrated that SH can synergistically enhance the efficacy of RAI through apoptosis, DNA damage repair, and cell cycle checkpoint regulation [20]. Combined with the redifferentiation effect of SH in this study, this provides some valuable evidence that SH may become an adjuvant treatment for PTC in RAI therapy.

Despite our finding that SH increased iodide-handling genes in PTC, some issues should be addressed. We found that SH can increase the uptake of TSHR-dependent RAI, but the specific mechanism that causes the increase in TSHR remains to be explored. Additional in vivo studies are necessary to conclusively confirm their responsiveness to RAI therapy in thyroid cancer patients. In conclusion, SH may serve as a potential treatment for PTC, especially for RAIR-DTC, by restoring NIS expression and the iodide uptake capacity. Therefore, considering the advantages of SH as natural, economical, and safe, SH treatment emerges as a promising adjunctive therapy for thyroid cancer patients and especially, for RAI-refractory thyroid cancer in the future.

## 4. Materials and Methods

### 4.1. Cell Culture and Treatment

The BCPAP and TPC-1 cell lines were provided by Professor Peng Hou (Key Laboratory for Tumor Precision Medicine of Shaanxi Province, Xi’an, China). The BCPAP cells and the TPC-1 cells were cultured in RPMI 1640 medium (Gibco, Carlsbad, CA, USA) and DMEM/F12 medium (Gibco, Carlsbad, CA, USA), respectively, containing 10% fetal bovine serum (FBS; Biological Industries, Kibbutz Beit Haemek, Israel) and 1% penicillin/streptomycin (Sigma-Aldrich, St. Louis, MO, USA) in a 5% CO_2_ humidified atmosphere at 37 °C.

A 50 mM stock solution was prepared by dissolving SH (Shanghai Yuanye Bio-Technology Co., Ltd., Shangai, China) in phosphate buffered saline (PBS; Biological Industries, Kibbutz Beit Haemek, Israel) and was stored at −20 °C for up to one month. SH-containing medium was refreshed every 48 h. For some experiments, the adenylyl cyclase inhibitor SQ22536 (MCE, South Brunswick Township, NJ, USA) was added 1 h before SH treatment in medium containing 2% FBS.

### 4.2. Cell Proliferation Assay

Cell counting kit-8 (CCK-8; Dojindo, Kyushu, Japan) assay was used to assess cell viability according to manufacturer instructions. The BCPAP and the TPC-1 cells were seeded into 96-well culture plates at 4000 cells/well for 24 h. Then, cells were treated with different doses of SH (0, 1, 2, 4, 6, 8 mmol/L) for 12 h, 24 h, and 48 h. After the treatments, CCK8 reagent was added into each well, and the cells were incubated at 37 °C for 2 h. The colored solution was quantified using a spectrophotometer at an absorbance of 450 nm. All samples were assayed in triplicate, and the mean for each experiment was calculated. Data were expressed as fold increases over control.

### 4.3. Apoptosis Assay

The BCPAP and the TPC-1 cells were seeded into 6-well culture plates at 50,000 cells/well for 24 h. Cells were treated with different doses of SH (0, 1, 2, 4, and 6 mmol/L) for 24 h. Apoptosis assay was measured using flow cytometry and the annexin V-7AAD apoptosis detection kit (Dalian Meilun Biotechnology Co., Ltd., Dalian, China). At least 1 × 10^6^ cells were incubated at 4 °C with propidium iodide and annexin V-7AAD, and the percentage of apoptotic cells was calculated using flow cytometry (BD Biosciences, Franklin Lakes, NJ, USA).

### 4.4. RNA Extraction and Reverse Transcription Quantitative Polymerase Chain Reaction (RT qPCR)

Total RNA was extracted from cells with TRIzol reagent (Life Technologies, Carlsbad, CA, USA) according to manufacturer instructions. Total RNA (1000 ng) was converted to synthesized cDNA using the first strand cDNA synthesis kit (Takara, Osaka City, Japan) in a reaction volume of 20 μL. Glyceraldehyde-3-phosphate dehydrogenase (GAPDH) was used as the internal control. Following reverse transcription, RT-qPCR was run on a Roche LightCycler 96 real-time fluorescence quantitative PCR instrument (Roche Diagnostics GmbH, Mannheim, Germany) using the SYBR Green (Takara, Osaka City, Japan). Primers for NIS, TG, TPO, TSHR, and PAX8 were used to analyze changes in mRNA expression levels of thyroid iodine-handling gene after treatment with SH. The 2^−ΔΔCt^ RT-qPCR analysis method was used to calculate relative expression levels of the target genes. The primers used in these experiments are listed in Table S1. All RT-qPCR experiments were performed in triplicate.

### 4.5. Western Blotting

Cells were lysed in RIPA buffer (Beyotime, Shanghai, China). All protein extracts were denatured in a boiling water bath (100 °C) for 6 min except for NIS, which was heated at 37 °C for 25 min. Membrane and cytoplasmic proteins were extracted from the soluble protein fraction using a Mem-PER^TM^ Plus kit (Thermo Fisher Scientific, Waltham, MA, USA) according to the manufacturer protocol. Equal amounts of protein were separated on 8% and 10% sodium dodecyl sulfate-polyacrylamide gel. After electrophoresis, proteins were transferred onto PVDF membranes (Millipore, Burlington, MA, USA) and blocked for 1.5 h with 5% BSA at room temperature. Then, the membranes were incubated at 4 °C overnight with the following primary antibodies: anti-NIS (1:500; Abcam, Cambridge, UK), anti-TSHR (1:500; Proteintech, Wuhan, China), anti-cAMP (1:20,000; Abcam, Cambridge, UK), anti-CREB (1:500; Abcam, Cambridge, UK), anti-PAX8 (1:500; Proteintech, Wuhan, China), anti-GAPDH (1:10,000; Proteintech, Wuhan, China), and anti-ATP1A1 (1:5000; Proteintech, Wuhan, China). After washing with TBST buffer five times, the membrane was incubated with secondary anti-rabbit or -mouse immunoglobulin (1:10,000; Proteintech, Wuhan, China) for 1 h at room temperature. Protein bands in the membrane were visualized with ECL reaction reagents (Millipore, Burlington, MA, USA), and the quantitative analysis of band intensity was performed by ImageJ software (Supplementary Materials S1).

### 4.6. Immunofluorescence

The BCPAP and TPC-1 cells were seeded onto 12 mm glass coverslips in a 24-well plate at 1.0 × 10^5^ cells/well. After treatment with SH (0, 4 mM) for 48 h, they were fixed with 4% paraformaldehyde for 15 min, then permeabilized using 0.5% Triton X-100 and blocked with 5% BSA for 30 min at room temperature. Cells were incubated in anti-NIS antibody (1:50; Abcam, Cambridge, UK) overnight at 4 °C and then incubated with Alexa Fluor 488-conjugated goat anti-rabbit IgG secondary antibody (1:200; Zhuangzhi Bio, Xi’an, China) in the dark for 1.5 h at room temperature. Nuclei were stained with DAPI Fluoromount-G (SouthernBiotech, Birmingham, AL, USA). Images were observed using confocal fluorescence microscope (Leica TCS SP5, Heidelberg, German) to monitor NIS expression using high magnification fields (63×).

### 4.7. Iodide Uptake Assay

Iodide uptake assay was performed as previously reported [48]. The experiment was performed at the Department of Nuclear Medicine, the First Affiliated Hospital of Xi’an Jiaotong University. The BCPAP and TPC-1 cells were seeded into 12-well culture plates at 1.0 × 105 cells/well. After treatment with SH (0, 4 mM) for 48 h, the cells were washed with ice-cold modified Hanks’ balanced salt solution (HBSS) three times. The cells were incubated with 2 μCi Na125I in 5 mM non-radioactive NaI for 45 min at 37 °C. The cells were then washed with cold HBSS and lysed with 500 μL formic acid for 10 min. The radioactivity was measured in the cell lysates using a gamma radioimmunoassay counter (ANHUI USTC ZONKIA Scientific Instruments Co., Ltd., Hefei, China). For each experimental condition, some wells received 300 μM KClO4 (Sigma, St. Louis, MO, USA) for 30 min, a competitive NIS inhibitor, in order to determine the non-specific RAI uptake. The radioactivity was expressed as counts per minute (cpm)/10^6^ cells.

### 4.8. Statistical Analysis

All data are expressed as the mean ± standard deviation (SD), and statistical significance was determined by Student’s *t*-test using GraphPad Prism 5 (San Diego, CA, USA). The differences were considered to be statistically significant when *p* < 0.05.

## Supplementary Materials

The following supporting information can be downloaded at: https://www.mdpi.com/article/10.3390/ijms231810709/s1.
