# Supplementary material for: Sinomenine Hydrochloride Promotes TSHR-Dependent Redifferentiation in Papillary Thyroid Cancer
Source: Int J Mol Sci. 2022 Sep 14;23(18):10709. doi: 10.3390/ijms231810709 (PMC9500915; doi:10.3390/ijms231810709)
Supplement: Supplementary file 1 [file ijms-23-10709-s001.zip › Supplementary figures.pdf]

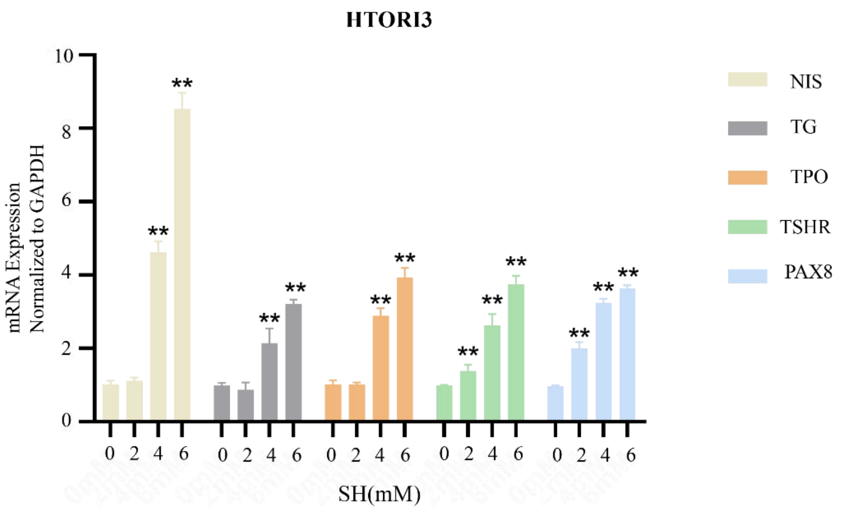

Figure S1. SH promoted cell redifferentiation in Htori3 cells. The Htori3 cells were exposed to fixed concentrations of SH (0,2,4,6mM) for 24 hours. SH upregulated the expression of thyroid-specific genes and the transcription factor expression. Data are the mean ± SD of three samples per group. ** p <0.005 vs. control. SH, sinomenine hydrochloride; NIS, sodium/iodide symporter; TG, thyroglobulin; TPO, thyroid peroxidase; TSHR, thyroid stimulating hormone receptor; PAX-8, paired box gene-8.

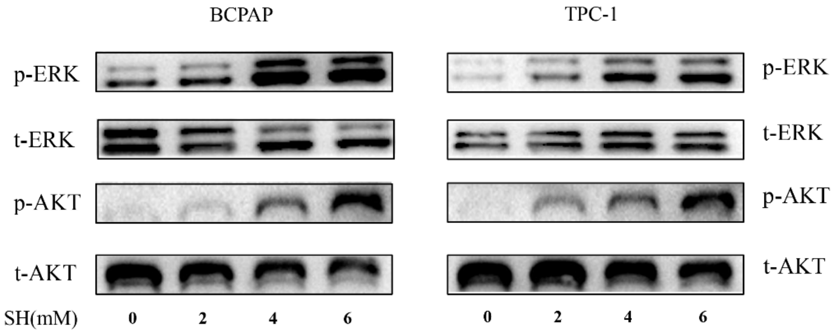

Figure S2. The expressions of p-ERK and p-AKT in BCPAP and TPC-1 cells were determined by western blot with quantitative analysis. Cells were treated with SH(0,2,4,6mM) for 48h. ERK and Akt phosphorylation were upregulated to various degrees. t-ERK and t-AKT was used as the loading control.
