# Supplementary material for: Sinomenine Hydrochloride Promotes TSHR-Dependent Redifferentiation in Papillary Thyroid Cancer
Source: Int J Mol Sci. 2022 Sep 14;23(18):10709. doi: 10.3390/ijms231810709 (PMC9500915; doi:10.3390/ijms231810709)

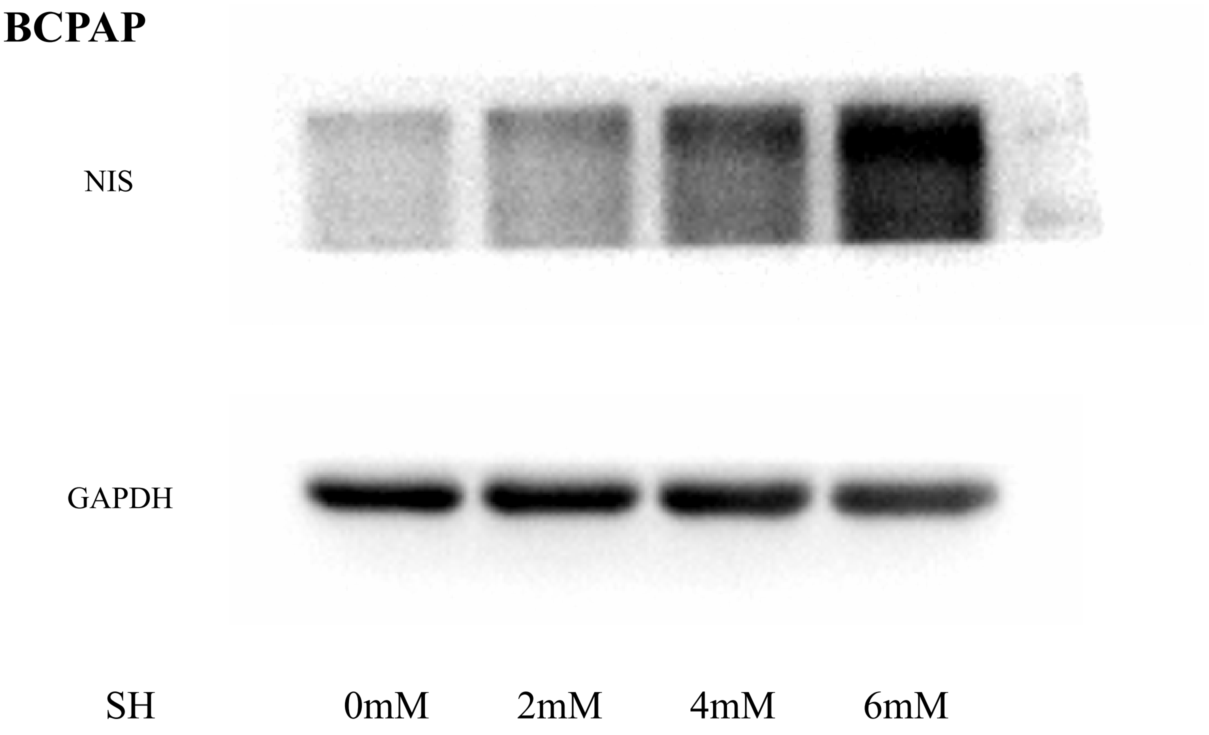
BCPAP
NIS
GAPDH
SH
0mM
2mM
4mM
6mM

**TPC-1**

NIS

GAPDH

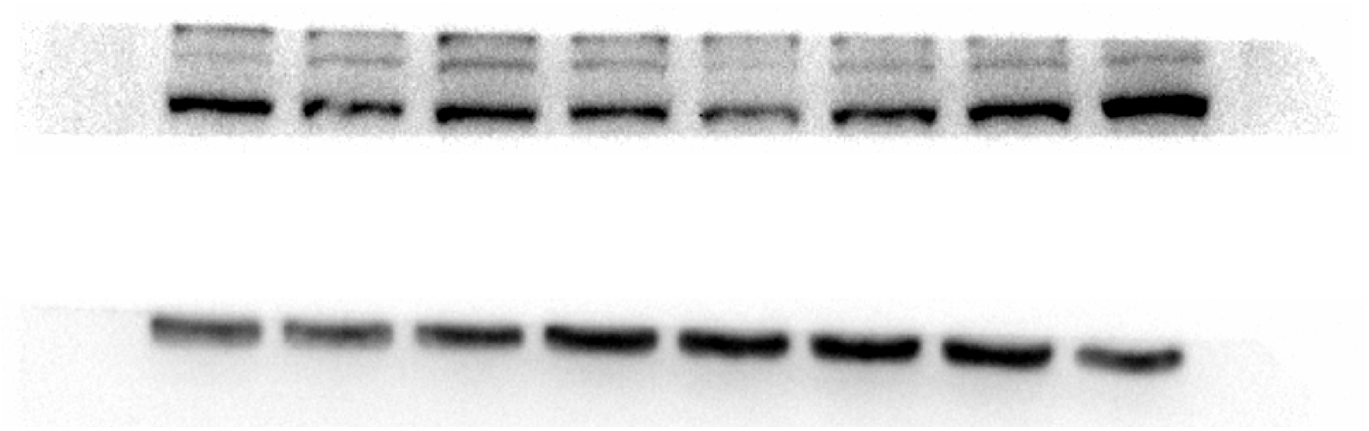

0mM 2mM 4mM 6mM SH

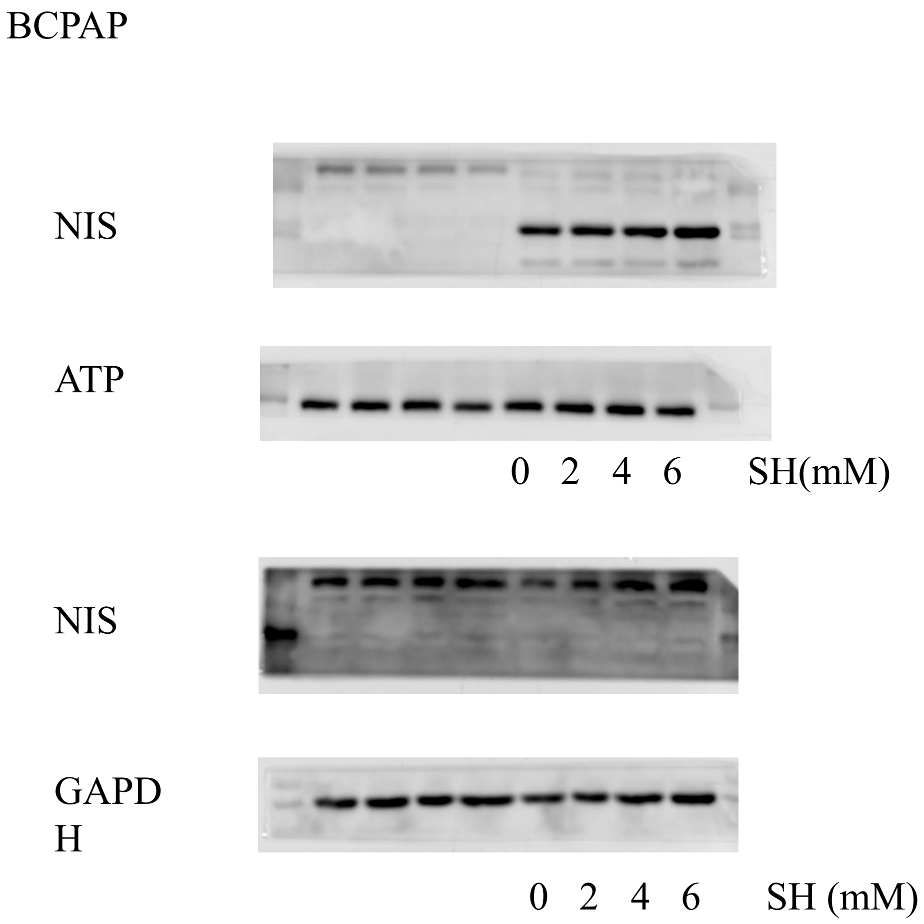
BCPAP
NIS
ATP
0 2 4 6 SH(mM)
NIS
GAPD
H
0 2 4 6 SH (mM)

TPC-1

NIS

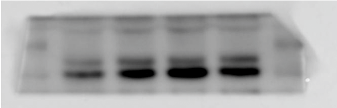

ATP

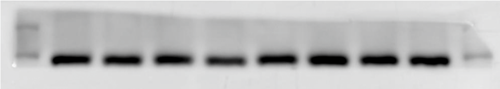

0 2 4 6 SH (mM)

NIS

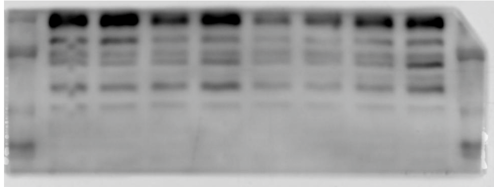

GAPD
H

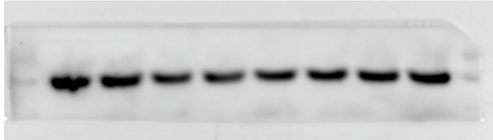

0 2 4 6 SH (mM)

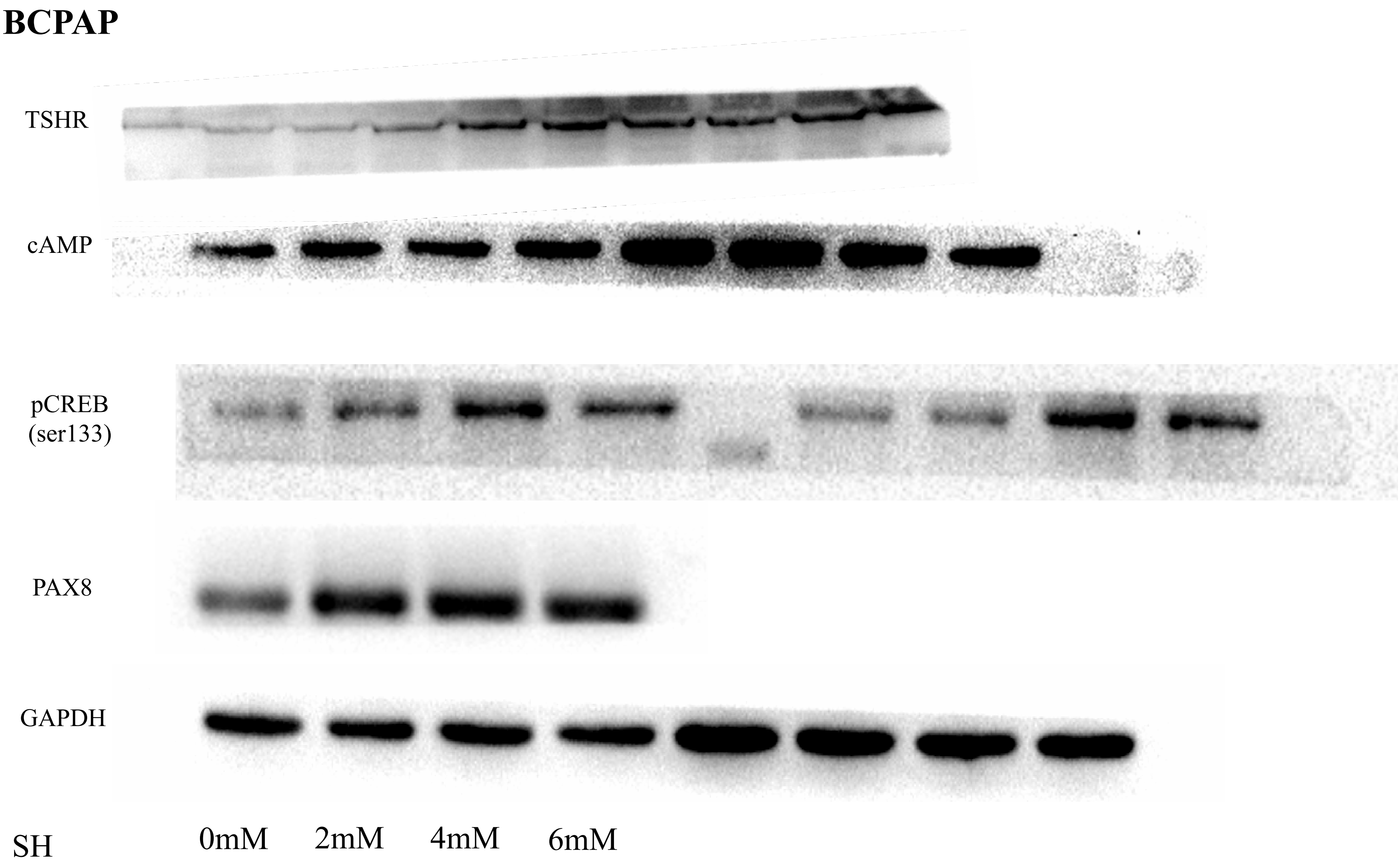
BCPAP
TSHR
cAMP
pCREB
(ser133)
PAX8
GAPDH
SH
0mM
2mM
4mM
6mM

**TPC-1**

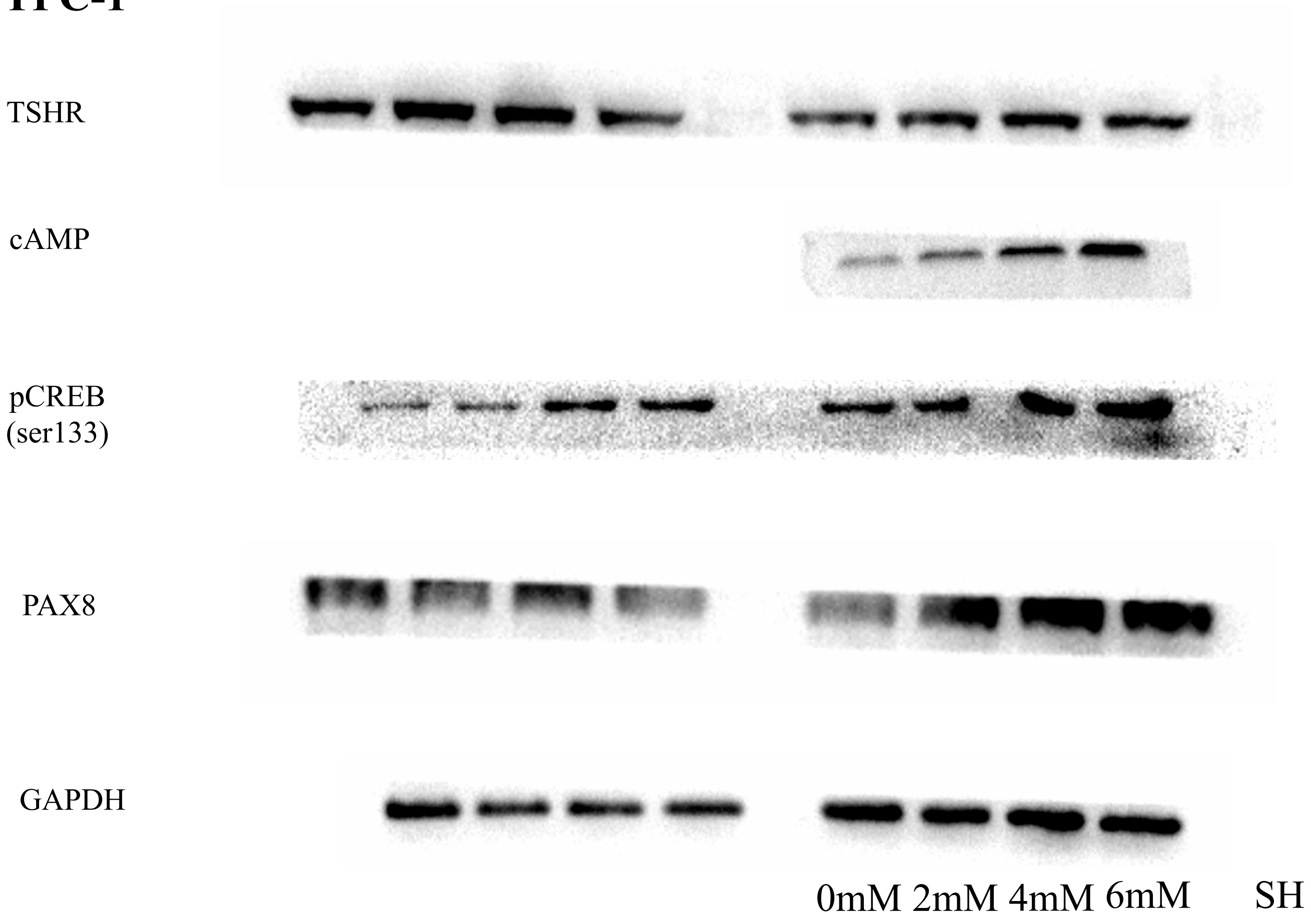

**BCPAP**

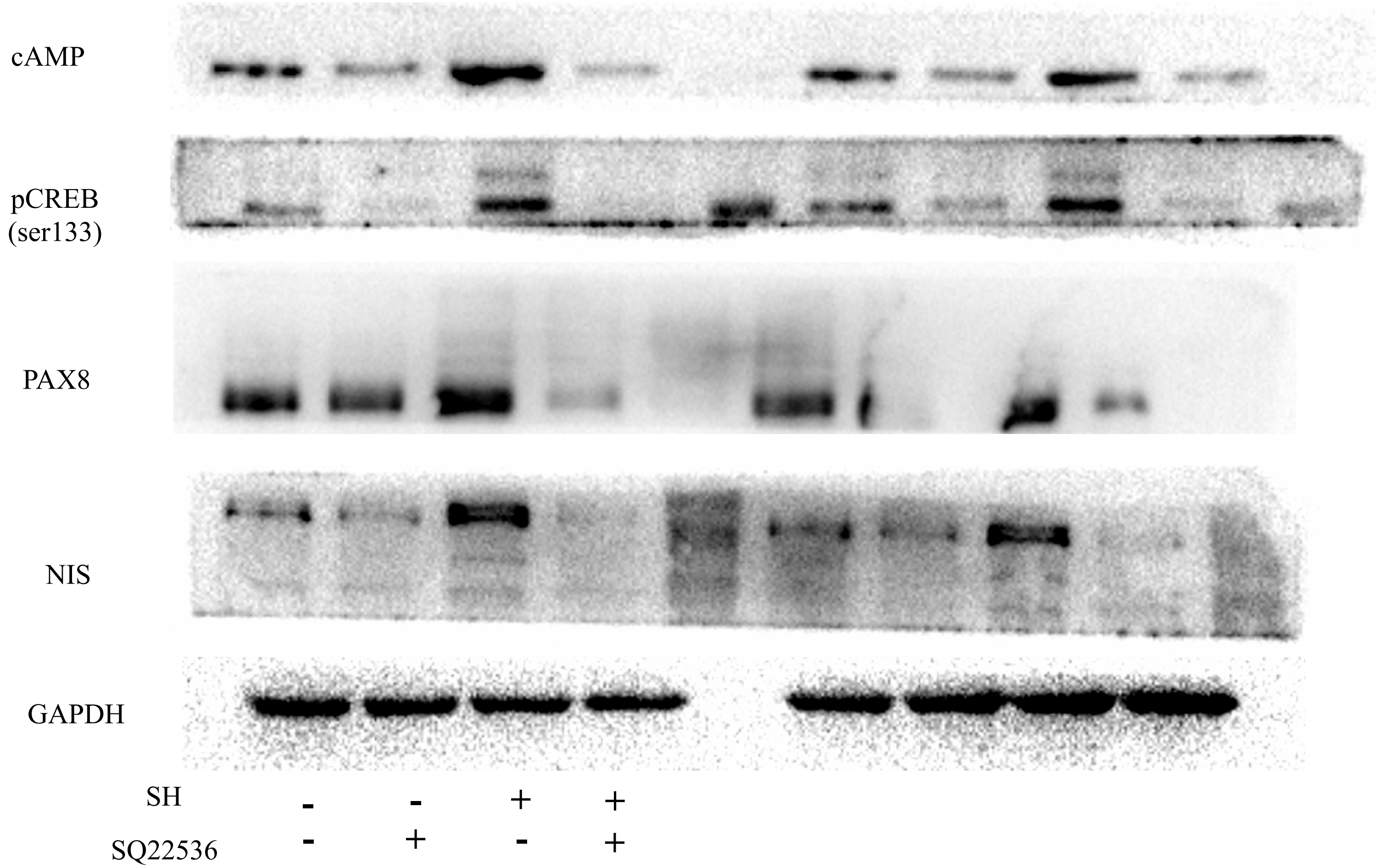

**TPC-1**

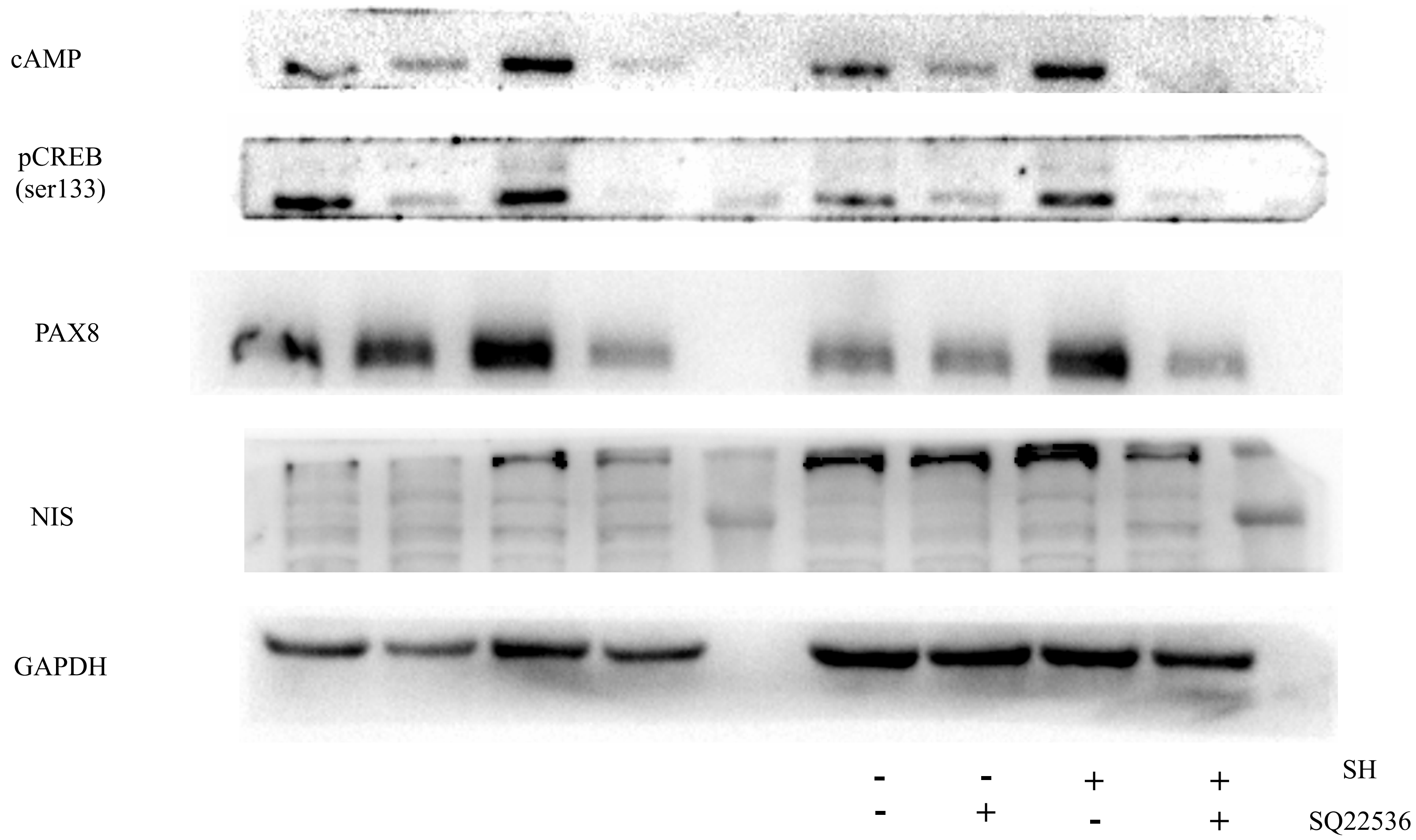

Supplement: Supplementary file 1 [file ijms-23-10709-s001.zip › Supplementary materials S1.pdf]
