# Supplementary material for: Sinomenine Hydrochloride Promotes TSHR-Dependent Redifferentiation in Papillary Thyroid Cancer
Source: Int J Mol Sci. 2022 Sep 14;23(18):10709. doi: 10.3390/ijms231810709 (PMC9500915; doi:10.3390/ijms231810709)
Supplement: Supplementary file 1 [file ijms-23-10709-s001.zip › Supplementary table.pdf]

Table S1. Primer sequences used in this study.

Gene	Forward (5′-3′)	Reverse (5′-3′)
NIS	TATCGCTATGGCCTCAAGTTCCTC	CTCCAGGTACTCGTAGGTGCTGGT
TG	CTGGCTGAGACAGGTTTGGA	GACTGATTGAACTGCGAGGAA
TPO	GGAGTCTCGTGTCTCTAGCGT	CTCTGCATCGTGGCGTACAT
TSHR	GGAATGGGGTGTTCGTCTCC	GCGTTGAATATCCTTGCAGGT
PAX8	TACTCTGGCAATGCCTATGG	TACAGATGGTCAAAGGCCG
GAPDH	CACTAGGCGCTCACTGTTCT	GCGCCCAATACGACCAAATC
